# Supplementary figures and images for: AvianLexiconAtlas: A database of descriptive categories of English-language bird names around the world
Source: PLoS One. 2025 Jun 11;20(6):e0325890. doi: 10.1371/journal.pone.0325890 (PMC12157040; doi:10.1371/journal.pone.0325890)

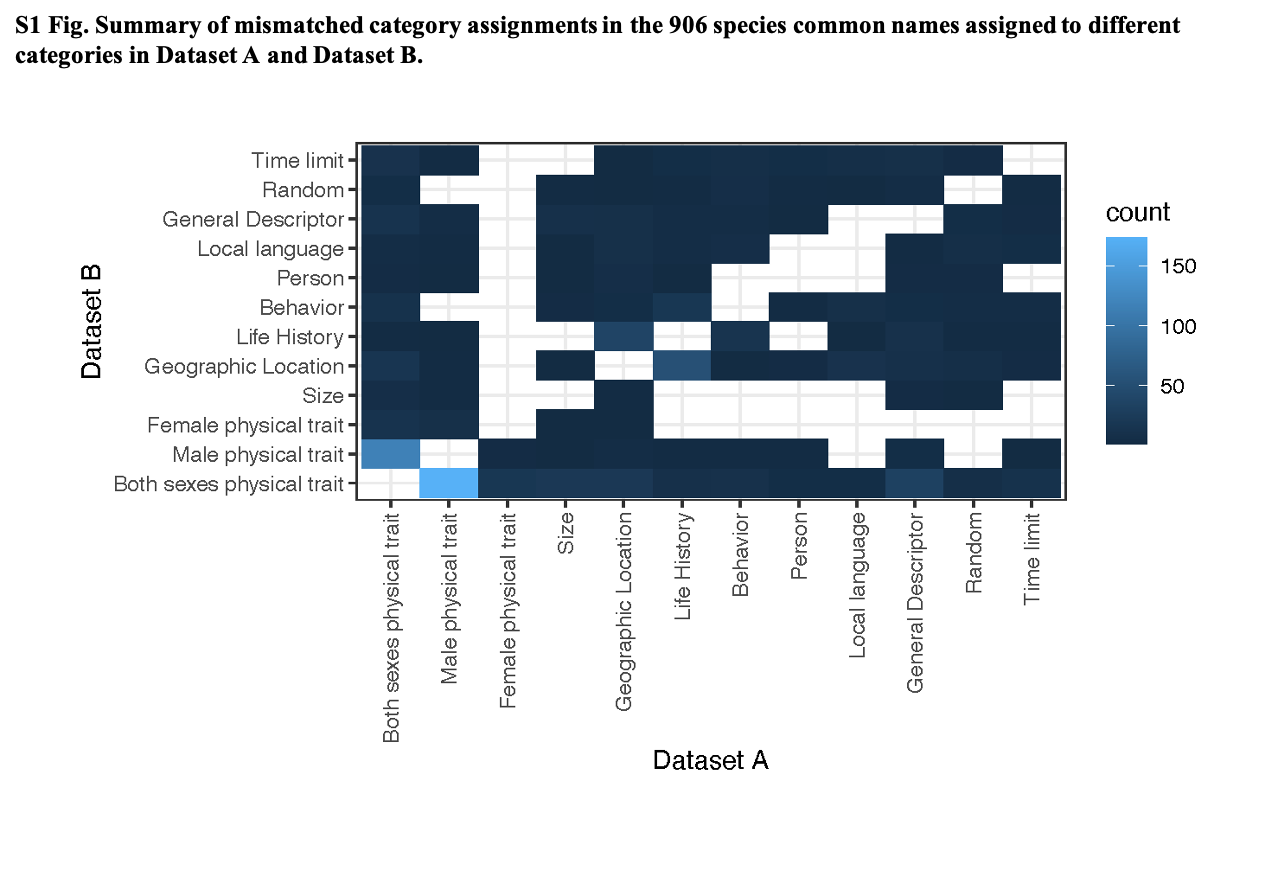

Supplement: S1 Fig — (TIF) [file pone.0325890.s001.tif]

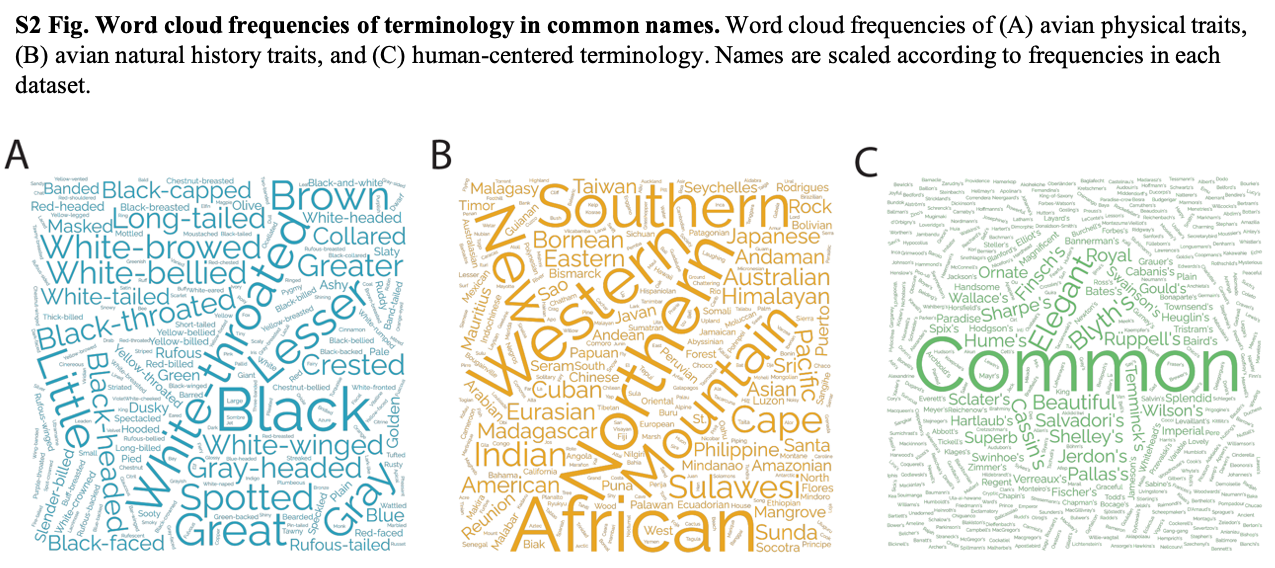

Supplement: S2 Fig — Word cloud frequencies of (A) avian physical traits, (B) avian natural history traits, and (C) human-centered terminology. Names are scaled according to frequencies in each dataset. (TIF) [file pone.0325890.s002.tif]

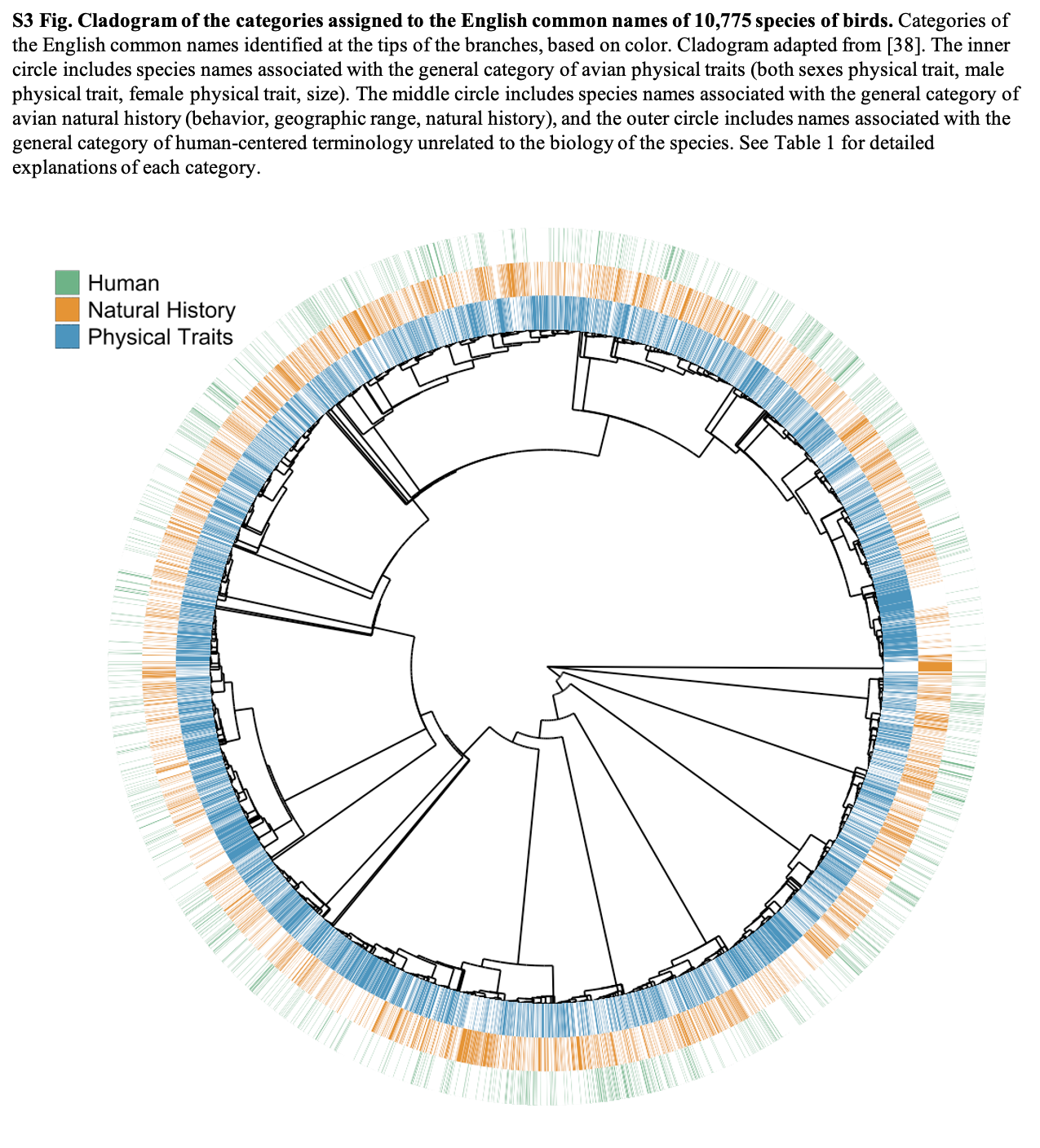

Supplement: S3 Fig — Categories of the English common names identified at the tips of the branches, based on color. Cladogram adapted from [38]. The inner circle includes species names associated with the general category of avian physical traits (both sexes physical trait, male physical trait, female physical trait, size). The middle circle includes species names associated with the general category of avian natural history (behavior, geographic range, natural history), and the outer circle includes names associated with the general category of human-centered terminology unrelated to the biology of the species. See Table 1 for detailed explanations of each category. (TIF) [file pone.0325890.s003.tif]
