# Supplementary material for: AvianLexiconAtlas: A database of descriptive categories of English-language bird names around the world
Source: PLoS One. 2025 Jun 11;20(6):e0325890. doi: 10.1371/journal.pone.0325890 (PMC12157040; doi:10.1371/journal.pone.0325890)
Supplement: S1 Table — Results of Fritz & Purvis’ [40] D statistic calculations for the phylogenetic structure of each of the grouped categories: physical traits, natural history, and human-centered terminology. For each grouped category, species assigned to the category were represented by a state of 1 and the remaining species assigned to other categories were represented by a state of 0. D is calculated by scaling the observed sum of sister-clade differences, Σdobs, with the mean values of the sum of sister-clade differences for 1,000 simulated trait distributions on the tips of the same phylogeny based on randomly reshuffling the trait values, Σdr , and trait evolution under Brownian motion Σdb: D = [Σdobs − mean(Σdb)/[mean(Σdr− mean(Σdb)]. An estimated D close to 1 represents a random distribution of a binary trait among related species on the phylogeny, while an estimated D close to 0 represents a clumped distribution of a binary trait among related species that would be expected under the Brownian motion model of evolution. Calculations were completed using the R package caper 1.0.3 [41]. (PDF) [file pone.0325890.s004.pdf]

S1 Table. Calculation of D statistics for the phylogenetic structure of categories. Results of Fritz & Pervis' [40] *D* statistic calculations for the phylogenetic structure of each of the grouped categories: physical traits, natural history, and human-constructed terminology. For each grouped category, species assigned to the category were represented by a state of 1 and the remaining species assigned to other categories were represented by a state of 0. *D* is calculated by scaling the observed sum of sister-clade differences, Σd_{obs}, with the mean values of the sum of sister-clade differences for 1,000 simulated trait distributions on the tips of the same phylogeny based on randomly reshuffling the trait values, Σd_r, and trait evolution under Brownian motion Σd_b: $D = [\Sigma d_{obs} - mean(\Sigma d_b)/[mean(\Sigma d_r) - mean(\Sigma d_b)]$. An estimated *D* close to 1 represents a random distribution of a binary trait among related species on the phylogeny, while an estimated *D* close to 0 represents a clumped distribution of a binary trait among related species that would be expected under the Brownian motion model of evolution. Calculations were completed using the R package *caper* 1.0.3 [41].

	**Physical Traits**	**Natural History**	**Human Terminology**
**Count of 0 states (no category assigned)**	4565	7361	9624
**Count of 1 states (category assigned)**	6210	3414	1151
**Observed sums of sister-clade differences, Σd_{obs}**	3757.153	3415.479	1761.211
**Mean sums of sister-clade differences of random reshuffling of traits, $mean(\Sigma d_r)$**	4560.358	4087.216	1906.918
**Mean sums of sister-clade differences of trait distributions under Brownian motion, $mean(\Sigma d_b)$**	1595.227	1451.21	733.921
**Estimated *D***	0.729	0.745	0.875
**Probability *D* significantly different from 1 (no phylogenetic structure)**	< 0.001	< 0.001	< 0.001
**Probability *D* significantly different from 0 (Brownian motion)**	< 0.001	< 0.001	< 0.001
