# Supplementary material for: AvianLexiconAtlas: A database of descriptive categories of English-language bird names around the world
Source: PLoS One. 2025 Jun 11;20(6):e0325890. doi: 10.1371/journal.pone.0325890 (PMC12157040; doi:10.1371/journal.pone.0325890)
Supplement: S1 Appendix — The data, glossary, and gazetteer reported in this article can be accessed at https://github.com/ajshultz/AvianLexiconAtlas. (PDF) [file pone.0325890.s005.pdf]

S1 Appendix. AvianLexiconAtlas Database Files. The data, glossary, and gazetteer reported in this article can be accessed at https://github.com/ajshultz/AvianLexiconAtlas.
