# Supplementary material for: AvianLexiconAtlas: A database of descriptive categories of English-language bird names around the world
Source: PLoS One. 2025 Jun 11;20(6):e0325890. doi: 10.1371/journal.pone.0325890 (PMC12157040; doi:10.1371/journal.pone.0325890)
Supplement: Attachment — Submitted filename: ResponsetoReviewers_PLOSONE_031725.pdf [file pone.0325890.s007.pdf]

NYU | ARTS & SCIENCE LIBERAL STUDIES

Erin S. Morrison, PhD
Clinical Assistant Professor

New York University | Liberal Studies
726 Broadway, 6th Floor
New York, NY 10003
erin.morrison@nyu.edu

March 17, 2025

Re: Resubmission of AvianLexiconAtlas: A database of descriptive categories of English-language bird names around the world (PONE-D-24-54740)

Dear Dr. Sugasawa,

Thank you and the two anonymous reviewers for the helpful feedback on our manuscript; we believe the comments have resulted in a considerably strengthened manuscript. We appreciate the opportunity to submit a revised version of the manuscript based on the reviews we received. Based on the main feedback we received from you and both reviewers, we have now reframed the manuscript to be a Research Article that solely reports on a new database, the AvianLexiconAtlas. We are no longer using the manuscript to present the results of original research that address a clearly defined research question. As part of this database description, we now include a section in the Results (lines 299-315) focusing on the implementation of the database that specifically provides a direct link to the database hosting site, plans for long-term database maintenance and growth. We retained the analyses included in the initial submission, but now provide support that these analyses are simply a way to validate the utility of the database. The trends that are presented in these analyses can be used to ask future questions about the utility of these English-language descriptors in a biological context as well as provide data for further examination into the linguistic history of these descriptors. In the Discussion we use these trends as a way to propose several areas of future research using the database.

As part of the revision, we have provided further details on why this study was exempt from the need for approval from the institutional review board. In the 'Data Collectors' section of the Methods (lines 194-201), we now include the full ethics statement. We specifically explain that the New York University (NYU) Human Research Protection Program (HRPP) determined via verbal and written consent that the work involved in the data collection for the database did not meet the criteria involving human subjects per the United States' regulations for the protection of human subjects (45CFR46.102(e)). This determination was made because the focus of the research was not on collecting information about the data collectors, but only involved a crowdsourcing model where the data collectors helped categorize the meanings behind unique descriptors in bird names. As such, the data collection methods did not require HRPP review and approval. We have included an official letter from the NYU Human Research Protection Program also stating this language in the revision documents. This has been submitted as an "Other" file. To further clarify that this study is a crowdsourced data collection research project associated with scientific nomenclature, and not a study on human subjects, we have changed from calling the individuals who helped with categorizing the bird names 'participants' and now call them 'data collectors' throughout the entire manuscript.

As per the journal's request, we now have an amended Role of Funder statement, and thank the journal editorial staff for changing this on our behalf:

"Funding was provided by the New York University Liberal Studies New Faculty Scholarship Award to E.S.M. The funders had no role in study design, data collection and analysis, decision to publish, or preparation of the manuscript."

Please note, that due to the removal of the S1 Table and the S1 Figure from the revised manuscript, the numbering of the remaining Supporting Information table and figures has now been changed. This has been updated in the manuscript, and we describe why this table and figure were removed from the Supporting Information documentation in detail below. We also edited the text for further clarity and *PLOS ONE* style requirements

Below is the list of specific changes incorporated in the revised manuscript:

1. **Reviewer 1:** *L91: It's not clear to me what "As part of these deliberations" refers to*

   Authors: This phrase has been deleted and in its place (lines 115-117) we have now clarified that the goal of constructing this database is to be able to understand what common names currently communicate about species in terms of the scope and variability of the terminology.

2. **Reviewer 1:** *L101-104: Why would you predict this? Much more context is needed to understand why there might be geographic and/or taxonomic patterns towards naming conventions.*

   Reviewer 1: *L104-106: What insights could this provide? This context is currently missing for the reader.*

   Reviewer 2: *I struggled to identify the main biological problem addressed in the manuscript or the main biological insight gained. I can understand the fact that name descriptors based on biology are more informative and that some name descriptors are very localised and so not very helpful for generalisation. I also understand that there are debates about fair naming – what bird common names shouldn't (or should) be. Is this analysis in light of these debates? Otherwise, if the main aim is to show how bird common names are arrived at or the unique histories of such, then the manuscript could be better focused, with that aim clearly stated, and more involving. There are some predictions outlined in the introduction and methods, like those associated with phylogenetic and geographical clustering of names (lines 100 – 104 and 230 - 231), but there isn't a clear indication of what might be the case if these predictions were right. Moreover, it might be important to show whether there are any biological implications for common names that do not follow accepted naming conventions (if any).*

   Authors: The predictions in the original manuscript in L101-104 have been deleted from the introduction to align with the focus of the paper on introducing the database. Since we are no longer presenting the work as a hypothesis test, we did not believe we should retain the predictions, which we believe led to some of the confusion about what the goal of this manuscript was. Instead, the introduction of phylogenetic and biogeographic analyses in the Introduction section (lines 125-132) are presented as a way to validate the utility of the

dataset for future research questions, and we propose several future research topics in the Discussion as part of our initial observations about the trends we observed in the database (lines 442-453).

3. **Reviewer 1:** *L485-486: This strikes me as an odd question, given that this manuscript presents data that can, in part, answer this question. Perhaps rephrase? In particular, what scientific * question * (ornithological, linguistic, ethno-ornithological, whatever) would be addressed by conducting an investigation like this?*

   Authors: We have added a line after this question to clarify its significance (lines 499-502).

4. **Reviewer 1:** *L147 onward: This is possibly an unpopular opinion, but I found this description of the 11-versus-10 category thing very hard to follow. It would perhaps be less confusing to either straight-up omit the mention of the 11 categories, or to say something like "Though 11 categories were initially scored, these were further refined during the data cleaning phase to a set of 10 categories [describe the 10]".*

   Authors: Thank you for pointing this out, we also can see how this could be confusing and per your advice have simplified the initial description of the categories in the Methods section (lines 171-177) and omit the mention of the 11 initial categories. We now only present the set of 10 categories used for terminology assignments. As part of this update, we moved the more detailed descriptions of each of the 10 categories into Table 1 that were previously only included in S1 Table. Since the S1 Table only existed to provide a comparison between the 11 initial categories and the 10 final categories we decided to delete the S1 Table about these 11 categories from the manuscript.

5. **Reviewer 1:** L152: *This may be rendered irrelevant by the above comment, but the use of "random" here is distracting, because of course birds do not have "random" names. They may have names that are miscellaneous, or opaque, or 'other', or something, but language is not 'random'.*

   Authors: We have addressed this issue by removing any mention of the initial 11 categories from the manuscript.

6. **Reviewer 1:** *Paragraph beginning L165: Were all data collectors fluent speakers of English? (Obviously for an undergraduate class, participants could not be excluded based on language ability, but it's useful context for a language-based coding task.)*

   Reviewer 1: *Similarly, for the scorers who made assessments based on photographs, did all scorers have typical colour vision (e.g., the ability to distinguish between red and green)?*

Authors: We have now added a clarifying sentence in the 'Data collection' subsection (lines 207-210) of the Methods that explains the use of duplicate datasets (in the first round of scoring) and triplicate datasets (in the second round of scoring) in the design of the data collection was established to validate the accuracy of the category assignments made by different people. We decided not to specifically address differences in English language fluency and/or color vision of the data collectors in the manuscript, because we did not ask any of the data collectors about this during the recruitment process.

7. **Reviewer 1:** *L180: Comma needed after the bracket/parenthesis.*

   Authors: The comma has now been added in line 204.

8. **Reviewer 1:** *L230: Again, why would you predict this? This needs to be spelled out somewhere (e.g., the introduction, or maybe the supplement).*

   Authors: Based on the decision to reframe the manuscript as a description of a new database, we have now changed the language in this section of the Methods and removed the mention of 'predictions' for the analyses used in the manuscript. Instead, this section is now called 'Data validation' (line 258) and we now described the analyses as a way to validate the utility of the database by simply exploring trends in the dataset that was collected (lines 259-296). This goal for the analyses has also been updated in the Introduction and Discussion sections to remove the implication we are hypothesis testing with these analyses.

9. **Reviewer 1:** *My understanding is that this phylogeny (and the associated R package) is currently unreviewed. I don't think *I* care (there's a trade-off to be had between including nearly all species in an unreviewed phylogeny and, say, losing ~10% of the species to use a published phylogeny with known biases, and I'm sympathetic to either decision), but the editor may have an opinion.*

   Authors: The decision to use the unpublished phylogeny that is currently in review was made because it most closely aligns with the taxonomy of the species in the database, because they are both based on the eBird/Clements taxonomy. This phylogeny, its documented methodology, and its associated R programs are all currently available as a preprint on *BioRxiv* (McTavish et al., https://www.biorxiv.org/content/10.1101/2024.05.20.595017v1). Additionally, as of the date of this letter the phylogeny has been cited by 4 published peer reviewed studies:

   - Barber, R.A. et al. (2024). *PLOS Biol.* 22(11): e3002856. https://doi.org/10.1371/journal.pbio.3002856
   - Janzen, E. & Etienne, R.S. (2024). *Mol Phylogenet Evol.* 200: 108168. https://doi.org/10.1016/j.ympev.2024.108168
   - Nussbaumer, R. et al. (2025). *Divers Distrib.* 31: e13935. https://doi.org/10.1111/ddi.13935

- Van Doren, B.M. et al. (2025). *Curr Biol*. 35(4): P898-904.E4. https://doi.org/10.1016/j.cub.2024.12.033

10. **Reviewer 1:** *Paragraph beginning L247: Why was this done as a chi-squared test and not with a phylogenetic correction? You know that there's a phylogenetic signal in the data, and that species naming conventions are non-independent – you should be correcting for that*

    Authors: Since several species did not have unique values for breeding locations, a phylogenetic regression analysis or a linear model was not possible. We agree that there may be some tendencies for species from the same families and orders to be named in a similar manner from the same geographic region, but in this manuscript we do not look to completely explain these patterns and we believe that question is now beyond the scope of the revised manuscript. Thus, we felt that the chi-squared test is describing patterns that may intrigue readers enough to investigate it more thoroughly elsewhere.

11. **Reviewer 1:** *L248: How did you extract the general region? How is "general region" defined? Also, is this in the published data and I just can't find it in the Github?*

    Authors: We provided more details in the "Data validation" section of the Methods to clarify what we mean by "extracted" the general region (lines 284-286). This data is compiled by the IOC World Bird List and we extracted the specific general regions for each species in the IOC World Bird list. We retained the definition for "general region" that was included in the initial submission of the manuscript, which describes the term as geographic ranges at the continent level, but we have now added a reference to the IOC World Bird List Range Terminology website so the reader can learn more details about how these designations were made. We have added the the general region or regions used for the breeding range of each species from the IOC World Bird List 13.2 to the 'total_dataset_allrounds.csv' file posted on the Github site for the database (S1 Appendix, https://github.com/ajshultz/AvianLexiconAtlas).

12. **Reviewer 2:** *Table 2: It took me a very long time to understand "Mean times repeated if not unique" and "Median times repeated if not unique". Please consider clarifying somewhere.*

    Authors: We have added text in the legend of Table 2 that clarifies that calculations for the mean and median frequencies of unique descriptors only include descriptors that occur more than once within species names in each category. Additionally, we changed the titles of the columns associated with the mean and median times to: "Mean frequency of unique descriptors" and "Median frequency of unique descriptors"

13. **Reviewer 1:** *Figure 2: How was this word cloud made? (What software/package?)*

Authors: We have now identified the R software package (wordcloud2) used to produce the word clouds in the "Data validation" section of the Methods (lines 262-263).

14. **Reviewer 1:** *L330: These differences in D values are relatively small; without confidence intervals, the reader doesn't know how to distinguish them. I'd suggest either adding confidence intervals, or softening the phrasing to "somewhat stronger" (or something).*

    Authors: The wording to describe the differences in the *D* values has been changed to "somewhat stronger" (line 358). There are no confidence intervals as part of the calculation for *D*, and we agree with Reviewer 1 that it is difficult to distinguish the magnitude of difference in this case

15. **Reviewer 1:** *Table S2: The last three rows of Table S2 are very difficult to understand unless you dig into Fritz & Purvis 2010 and look up the definitions of things like \sum d_{obs}. Consider either omitting or explaining somewhere how D is actually calculated?*

    Authors: Due to the deletion of the table that was originally S1 Table from the revised manuscript, the supporting information table reporting the calculations for the *D* statistic that was S2 Table in the original manuscript has now been changed to S1 Table in the revised manuscript. The legend of this table has now been expanded to provide more detail on how *D* is actually calculated. It includes the specific equation used for *D* along with clarifying definitions for each of the calculations and statistics reported in the table.

16. **Reviewer 1:**
    Gazette:
    • There are several changes in font and font size that you might want to fix.
    • You probably want to remove the note in red on the first page.
    • Do you have permission to publish these figures?
    • You might want to triple-check all of the political affiliations claimed in the geographic location section, and/or include a disclaimer; these in general seem excellent, but people have strong feelings about all sorts of things.
    • The Perijá entry is quite obviously copied from Wikipedia – you may want to disable the links and standardize the font, if nothing else.

    Authors: We have now standardized the font and font size in the document and removed the note in red on the first page. We have added a new note in red on the first page specifying the date the document was most recently updated. On the PDF of the Glossary & Gazetteer posted on the GitHub site we have included a link to the live Google Docs version of this file. We plan to start updating the database annually, and this will include updating the Glossary and Gazetteer. We did not have permission to publish the figures displaying the names and locations of field markers. These figures have been removed from the Glossary & Gazetteer and in their place we have included full citations and links for two reputable websites that host the image. To address the potential issues of misnomered geographic

locations, depending on cultural or political differences, we have added a disclaimer to the top of the Gazetteer that now states: "These descriptions are not meant to be exhaustive. Descriptions are based on what was needed to identify a term used in English common names for birds as an historic or current term associated with a geographic location. We recognize that the regions listed here may be associated with different terminology in different cultures and languages". Finally, the Perijá entry in the Gazetteer has been updated based on Reviewer 1's suggestions to standardize it with the rest of the entries.

17. **Reviewer 2:** *Authors have taken time (e.g line 273 - 275) to explain author contributions and criteria for qualifying for co-authorship. This is useful in some ways, but I do not believe that this is relevant for the main text. It might be required by the journal to judge fair authorship, but I believe that it should be included in the author contribution section, and not in the main text. If the inclusion or exclusion criteria has an impact on the results or the interpretation of it, then maybe these criteria discussed.*

    Reviewer 2: *Lines 417 – 501 discusses methodological considerations and participation. Down-sizing this aspect of the discussion may help make the manuscript better concise.*

    Authors: We have removed the Results section that presented comparisons of the contributions of authors to the database based on their level of expertise along with the criteria for co-authorship. This was made as part of the decision to reframe the manuscript to focus on the description of the database itself and introducing its utility for further research. Since the S1 Figure in the original manuscript summarized participation in the crowdsourced data collection based on the data collectors' expertise in ornithology, it was also removed from the revised manuscript.

    Following the decision to focus on the introduction of the database itself, we also removed the text that was in the in lines 417-501 of the original manuscript from the revised manuscript and chose not to focus any of the text on comparing scoring accuracies of data collectors with different levels of expertise. We still retained text that discusses, broadly, how accessible some of the English terminology was across all data collectors by discussing frequent category mismatches that occurred between duplicate datasets. We use this data to make the observation that many of the terms for trait descriptors, in particular, lack specificity.

18. **Reviewer 2:** *Line 109: it may be a goal of common names that they are useful for the general public, but they must not be generalised to the extent that they cease to make sense locally, except a species species means exactly the same thing to everyone, and I do not believe that this is the case. An interesting perspective for me would be to discuss how names differ according to context or locations (see you comment in lines 512 - 513).*

    Authors: We agree with this point, and in lines 503-531 of the Discussion we suggest several future research studies using the AvianLexiconDatabase that could investigate how common names differ according to context, locations, and across different languages.

19. **Reviewer 2:** *Lines 405 – species not specious*

    Authors: Specious has been changed to the term species-rich in line 435 to clarify the point we want to make in this sentence.

20. **Reviewer 2:** *The term human-constructed terminology is a bit misleading because all names are human-constructed including avian centred names.*

    Authors: To provide clarity on this broader category used only for descriptive analyses, we have changed the term 'human-constructed' to 'human-centered' throughout the manuscript.

21. **Reviewer 1:** *A lot of your discussion is really great. For example, your point about the naming of Grallaria species is a lovely insight, as is the idea that closely-related seabird species are more distinguishable by geography than by morphology. I'd, personally, love to see even more about the (highly colonial) history of how people have named birds in (American) English and how that interfaces with global patterns of, e.g., sexual dimorphism, community assembly, even if it's just anecdotal.*

    Reviewer 2: *your result which shows that Africa and Oceans show the highest proportions of species named after human-constructed terminology seemed to have been ignored in the discussion. Isn't it easily predictable that species described by colonial ornithologists (which may be the case for most of Africa and the Oceans) are more likely to be named after persons? You mentioned the outsized roles of western naturalists in lines 502 – 508. Although, I am curious about why South America has the least proportion of names based on human-constructed terminologies. The timing of species description may influence how they are described. Newly described species are less likely to have been described from a museum collection which you argue may have been the motivation for naming species based on physical traits.*

    Authors: These are all great suggestions for future research. We believe that digging further into the role that Western ornithologists have played in shaping the names of birds in geographic regions with a history of Western colonialism is beyond the scope of this revised manuscript, which now solely focuses on introducing the AvianLexiconDatabase as a resource to study questions just like these. In the revised Discussion, we make several suggestions for further historical and linguistic analyses of the distribution of the nomenclature categories in relation to phylogeny, biogeography, date of name assignment, and even the identity of the person the name is attributed to (lines 426-430, lines 442-453, lines 475-481, lines 498-502, lines 515-518, and lines 525-531). All of these would provide insight into how Western empire expansion and colonial perspectives have shaped the patterns observed in the database.

Thank you, again, to all of the reviewers for your insightful feedback on our initial manuscript submission and for taking the time to now review our revised manuscript. We believe this revision provides a much more focused presentation of the AvianLexiconAtlas and its future utility in interdisciplinary research.

Sincerely,

Dr. Erin Morrison and Dr. Allison Shultz
