# Supplementary material for: AvianLexiconAtlas: A database of descriptive categories of English-language bird names around the world
Source: PLoS One. 2025 Jun 11;20(6):e0325890. doi: 10.1371/journal.pone.0325890 (PMC12157040; doi:10.1371/journal.pone.0325890)
Supplement: Attachment — Submitted filename: ResponsetoReviewers2_PLOSONE_051525.pdf [file pone.0325890.s008.pdf]

NYU | ARTS & SCIENCE LIBERAL STUDIES

Erin S. Morrison, PhD
Clinical Assistant Professor

New York University | Liberal Studies
726 Broadway, 6th Floor
New York, NY 10003
erin.morrison@nyu.edu

May 15, 2025

Re: Revision of AvianLexiconAtlas: A database of descriptive categories of English-language bird names around the world (PONE-D-24-54740R1)

Dear Dr. Sugasawa,

Thank you and the two anonymous reviewers for taking the time to review our revised manuscript and providing further feedback. We appreciate the opportunity to continue to revise the manuscript, and believe we have thoroughly addressed the comments from the reviewers.

As part of the first revision, we provided further details on why this study was exempt from the need for approval from the institutional review board. As requested by the journal, we have included an Ethics statement subsection in lines 145-153 at the start of the Materials and methods section. We specifically explain that the New York University (NYU) Human Research Protection Program (HRPP) determined via verbal and written consent that the work involved in the data collection for the database did not meet the criteria involving human subjects per the United States' regulations for the protection of human subjects (45CFR46.102(e)). This determination was made because the focus of the research was not on collecting information about the data collectors, but only involved a crowdsourcing model where the data collectors helped categorize the meanings behind unique descriptors in bird names. As such, the data collection methods did not require HRPP review and approval. We have included an official letter from the NYU Human Research Protection Program also stating this language in the revision documents. This has been submitted as an "Other" file. To further clarify that this study is a crowdsourced data collection research project associated with scientific nomenclature, and not a study on human subjects, we have changed from calling the individuals who helped with categorizing the bird names 'participants' and now call them 'data collectors' throughout the entire manuscript.

As per the journal's request, we now have an amended Role of Funder statement, and thank the journal editorial staff for changing this on our behalf:

"Funding was provided by the New York University Liberal Studies New Faculty Scholarship Award to E.S.M. The funders had no role in study design, data collection and analysis, decision to publish, or preparation of the manuscript."

Below is the list of specific changes incorporated in the revised manuscript:

1. **Reviewer 2 & Academic Editor:** *Reviewer2 commented that the discussion section should clarify the database utility and prospects, which I agree. As the current discussion is structured around the results and the constraints of the database, the significance and future ideas for the database are scattered across the discussion, making it hard to get a comprehensive and concrete idea of how this database contributes to research in biology and other fields. Please clarify the utility and future prospects of the database in discussion -- I*

think that rewriting the concluding paragraph might be the least destructive way, but am open to other ways to achieve this.

Authors: We appreciate the comments by Reviewer 2 and the Academic Editor, and have revised the Discussion as suggested. We now have restructured the end of the discussion to be centered on the utility and future prospects of the database. We have reframed the constraints as opportunities to expand the database in the future, summarize all of the opportunities for future research directions mentioned throughout the Discussion, and end on highlighting the significance of the AvianLexiconAtlas for both professional researchers and amateurs.

2. **Reviewer 1:**
   L74: They're not necessarily Latin. "Latinized"? "binomial, often in Latin"? Something else?
   L103: Perhaps the "gull" family of birds? Or just "gull species"?
   L238: Purvis, not Pervis

   Authors: In line 100, we have edited this passage to now say that ICZN scientific names are written as a binomial, often in Latin. In line 276 we corrected the spelling error and it now says Purvis.

   The topics of the lines referenced by Reviewer 1 don't match the ones in the manuscript file we submitted in the first revision. In the case of the referenced line 103, we were not entirely sure what this was in reference to, as we don't mention any gull species in the manuscript. We thought the comment might be referring to the use of "shared group names" to describe the part of a species' English common name that is shared across species (for example Ostrich) in lines 160-165 in the manuscript for the first revision. In the current version of the manuscript, we have now deleted the term "group" in lines 172-177, and instead refer to the names that occur in multiple species, as "shared names". We chose not to specifically define these shared names as 'family' names, because the shared descriptor across species may not always correspond directly to taxonomic groups.

3. **Reviewer 1:** *L248-249: What are some other general regions, if not continents?*

   In lines 288-291 we have now clarified that the general range classifications for species occur at the subcontinent level (instead saying that the classifications were generally, but not always, at the continent level) and have added that in addition to these regions there is a separate classification for primarily oceanic species.

4. **Reviewer 1:** *L537-538: This is extremely picky, but I'd recommend phrasing this sentence with a little less surprise; the fact that colour naming varies among people is an extremely well-documented anthropological phenomenon (e.g., https://doi.org/10.1073/pnas.2109237118 , or even Berlin & Kay's "Basic Color Terms")*

   Authors: Thank you for bringing this work to our attention. We have now incorporated both of these sources into the manuscript in lines 474-478 to hypothesize that the diversity of

color terms observed in the dataset are likely associated with how often general categories of color are referenced across species. This section about color terminology was moved from the concluding paragraph of the Discussion to an earlier paragraph in the Discussion that provides an overview of the issues data collectors faced with unfamiliar terminology.

5. **Reviewer 1:** *Figures – as a heads-up, Manuscript Central (or whatever software this is – "Editorial Manager"??) has compressed your figures in a weird way. It's also obliterated the mathematical formatting on Page 35.*

   Authors: Thank you for bringing this to our attention. We have been in touch with the PLOS ONE editorial office and were told that "the reduction in resolution is part of the PDF building process that cannot be prevented, and the PDF version of manuscripts will always contain a compressed version of very high-resolution figures." The editorial office advised us that all of the files are available to download in their original format in the manuscript's file inventory. We tested this and the files and images were viewable in the form we intended.

6. **Reviewer 1:**
   Gazetteer:
   • Extra square bracket under 'Dimorphic'
   • Extra bolding in 'pinnated', 'russet', 'tawny'
   • I don't know what "If toponym varies from spelling indexed under, listed at end of entry" means?

   Authors: The extra square bracket and extra bolding in those entries have now been removed. We have now reworded the phrase *"If toponym varies from spelling indexed under, listed at end of entry"* that was located at the start of the Gazetteer to clarify its meaning, and it is now phrased as: *"Index of specific place names. Any documented variations of the spelling for an indexed place name are listed at the end of its entry."* The new version of the Glossary and Gazetteer file has been uploaded to the GitHub site.

7. **Authors:** In the first round of feedback we received, there was concern that the phylogeny referenced in the manuscript was currently unreviewed. In the time since we submitted the first revision, however, the phylogeny has now been peer reviewed and published in *PNAS*:

   McTavish EJ, Gerbracht JA, Holder MT, Iliff MJ, Lepage D, Rasmussen PC, et al. A complete and dynamic tree of birds. Proc Natl Acad Sci. 2025;122(18):e2409658122. https://doi.org/10.1073/pnas.2409658122

   We have now updated the citation (38) for the phylogeny in the revised manuscript to its peer reviewed publication.

Thank you, again, to all of the reviewers for your continued feedback.

Sincerely,

Dr. Erin Morrison and Dr. Allison Shultz
